# Involvement of Microglia in Neurodegenerative Diseases: Beneficial Effects of Docosahexahenoic Acid (DHA) Supplied by Food or Combined with Nanoparticles

**DOI:** 10.3390/ijms221910639

**Published:** 2021-09-30

**Authors:** Karine Charrière, Imen Ghzaiel, Gérard Lizard, Anne Vejux

**Affiliations:** 1 Centre Hospitalier Universitaire de Besançon, Centre d’Investigation Clinique, INSERM CIC 1431, 25030 Besançon, France; karine.charriere@gmail.com; 2 Team Bio-PeroxIL, “Biochemistry of the Peroxisome, Inflammation and Lipid Metabolism” (EA7270), Université de Bourgogne Franche-Comté, INSERM, UFR Sciences Vie Terre et Environnement, 21000 Dijon, France; imenghzaiel93@gmail.com (I.G.); gerard.lizard@u-bourgogne.fr (G.L.)

**Keywords:** docosahexaenoic acid, microglia, neurodegenerative disease, inflammation, nanomedicine

## Abstract

Neurodegenerative diseases represent a major public health issue and require better therapeutic management. The treatments developed mainly target neuronal activity. However, an inflammatory component must be considered, and microglia may constitute an important therapeutic target. Given the difficulty in developing molecules that can cross the blood–brain barrier, the use of food-derived molecules may be an interesting therapeutic avenue. Docosahexaenoic acid (DHA), an omega-3 polyunsaturated fatty acid (22:6 omega-3), has an inhibitory action on cell death and oxidative stress induced in the microglia. It also acts on the inflammatory activity of microglia. These data obtained in vitro or on animal models are corroborated by clinical trials showing a protective effect of DHA. Whereas DHA crosses the blood–brain barrier, nutritional intake lacks specificity at both the tissue and cellular level. Nanomedicine offers new tools which favor the delivery of DHA at the cerebral level, especially in microglial cells. Because of the biological activities of DHA and the associated nanotargeting techniques, DHA represents a therapeutic molecule of interest for the treatment of neurodegenerative diseases.

## 1. Introduction

Neurodegenerative diseases represent a major public health issue in the world. Indeed, due to the progressive aging of the population and the lack of curative treatments, the number of people suffering from neurodegenerative diseases has increased considerably in recent years and is expected to continue to grow steadily in the years to come. These pathologies are chronic progressive diseases that affect the central nervous system, mainly the neurons that are often the target of therapies. The causes of these pathologies are to be found in genetics or risk factors such as the presence of chemical molecules in food, air, water, houses, and everyday objects. These different risk factors can contribute to oxidative stress, inflammation, and peroxisomal and mitochondrial dysfunctions, ultimately leading to neuronal death [1]. Most therapies target neurons and their function. However, there is an inflammatory component that must also be considered, and thus the involvement of microglia, in these pathologies [2]. Microglial cells are the major resident immune cells in the brain. Microglia activation is often classified into two opposite states: M1 and M2 [3]. The M1 state corresponds to a “classical activation” and is considered to be proinflammatory with a high capacity to present antigens, and high production of nitric oxide (NO) and reactive oxygen species (ROS) as well as pro-inflammatory cytokines. The M2 state includes both “alternative activation” and “acquired deactivation” and expresses the phenotypic markers arginase-1 (Arg1), CD206, interleukin (IL)-10, transforming growth factor β (TGF-β), and IL-1. This M2 state is considered to be an anti-inflammatory state [4] with the capacity to fine-tune inflammation, debris removal, promotion of angiogenesis, and tissue remodeling and repair. This separation into two opposite states does not reflect all the microglia phenotypes which will depend on the brain injury, its stage, and its location [2,5]. Nevertheless, microglial cells can be neuroprotective or neurotoxic depending on their activation status and the M1/M2 terminology remains useful to describe these two properties, while keeping in mind that it does not allow all reactive states of microglia to be described.

In physiological conditions, microglial cells work as sentinels. When they are activated by injurious stimuli, they can turn into several phenotypes, the two main ones being M1 and M2.

M1 produces pro-inflammatory cytokines that allow neuroprotection by removing pathological agents or recruiting additional cells. In this case, neuro-inflammation caused by microglia is neuroprotective. In contrast, a prolonged neuro-inflammation induces neurotoxicity and leads to neurodegeneration [6]. Briefly, M1 cells act as potent effectors that drive the inflammatory response, can have detrimental effects on neural cells, and participate in neuronal cell death if the switch to the M2 state does not occur in an appropriate time frame [7].

Therefore, modulating the activation state of microglia and their tendency toward the M2 state could be a promising therapeutic approach for central nervous system repair and regeneration. 

Given the lack of effective treatment and the difficulty of developing a molecule capable of crossing the blood–brain barrier (BBB), the use of food-derived molecules has been raised as a possible therapeutic option to target the current inflammatory state or to improve the phagocytic activity of microglia. Among these molecules, docosahexaenoic acid (DHA; C22:6 omega-3), an omega-3 polyunsaturated fatty acid, has many advantages. 

In this review, we will focus on the potential cytoprotective effects of DHA on oxidative stress, cell death affecting microglia, and on microglia-controlled inflammation. We will also present some human clinical trials that show the benefits of using DHA to improve the therapeutic management of patients with neurodegenerative diseases along with certain limits to the use of DHA in capsules or from food. Finally, we will present different approaches using nanoparticles that could allow for a better availability of DHA at the cerebral level.

## 2. DHA Biochemistry

**Figure 1:**
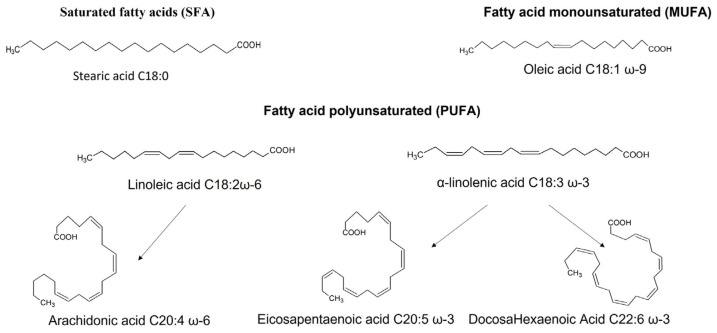
The different families of fatty acids and their main members. Fatty acids are classified according to the saturation or unsaturation of the carbon chain. There are saturated fatty acids (SFA), monounsaturated fatty acids (MUFA), and polyunsaturated fatty acids (PUFA). Among the latter, we distinguish between omega-3 and omega-6. Docosahexaenoic acid belongs to the omega-3 family.

DHA belongs to the family of fatty acids (FA) which are divided into distinct families according to the amount of carbon they contain and the presence or absence of unsaturations in their hydrocarbon chain (Figure 1) (see review [8]). Thus, we find saturated fatty acids (SFA) that have no double bond, the most common of which are palmitic acid (C16:0) and stearic acid (C18:0). Then, the presence of a single double bond in the carbon chain qualifies the fatty acid as monounsaturated (MUFA); this is the case for oleic acid (C18:1 omega-9). Finally, from two double bonds, we have polyunsaturated fatty acids (PUFA). This last family is divided into two subfamilies: the omega-6 family and the omega-3 family, which are distinguished by the position of the first double bond carried, respectively, by the sixth (n-6 or ω6) or third carbon (n-3 or ω3) from the terminal methyl end. PUFAs 6 and 3 (C20 and C22) are derived from two indispensable precursors, respectively: linoleic acid and α-linolenic acid (consisting of 18 carbon atoms and 3 double bonds).

**Figure 2:**
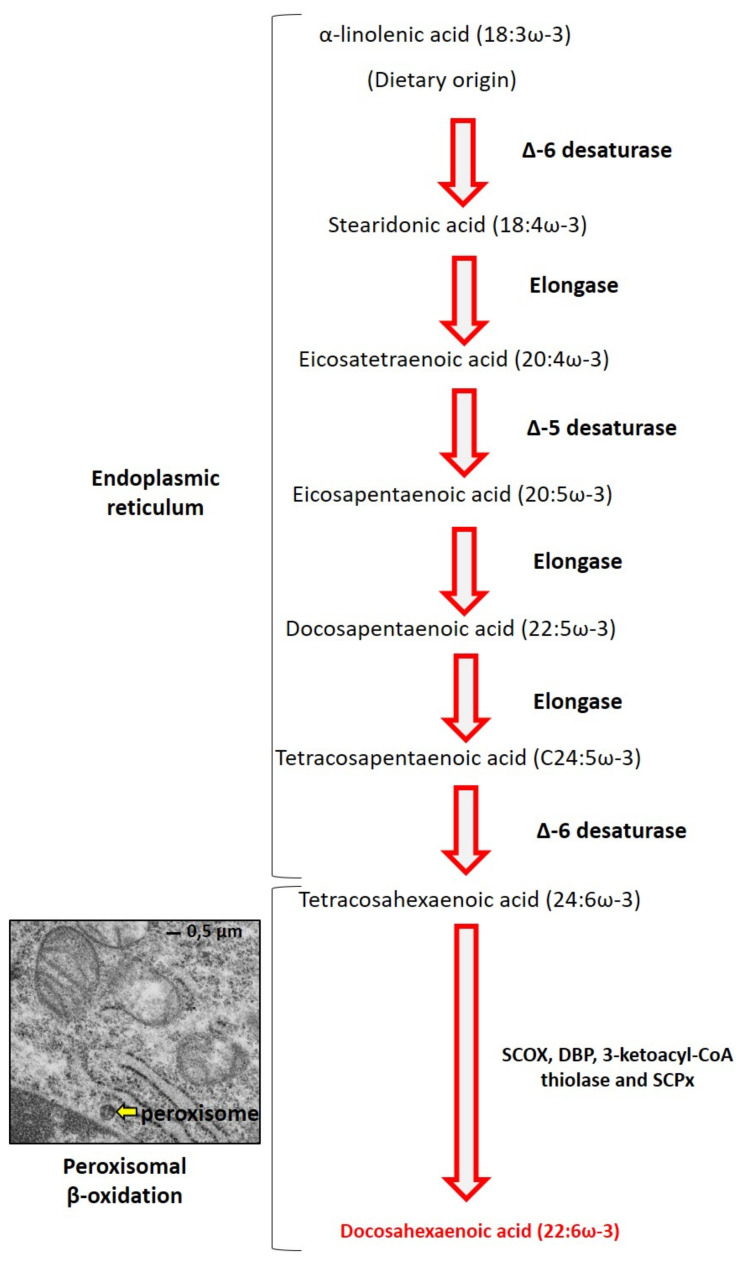
Biochemical pathway of DHA biosynthesis. In the endoplasmic reticulum, α-linolenic acid is converted to tetracosahexaenoic acid by the action of elongases and desaturases. Then, in the peroxisome (yellow arrow: electron microscopy of C2C12 myoblasts; provided by Imen Ghzaiel, PhD student), β-oxidation occurs and leads to the synthesis of DHA.

DHA is a major component of PUFAs in all cell membranes. It is necessary for proper development of the retina and the central nervous system (CNS) [9]. DHA deficiency disrupts the composition membrane lipid and thus the functions of astrocytes at the CNS level [10]. DHA is synthesized in the organism from essential precursors (α-linolenic acid C18:3 ω-3) provided by food [11]. The formation of DHA involves a succession of desaturations and elongations, which take place mainly in the liver, muscles, or even adipose tissue (Figure 2) [12]. DHA is synthesized from α-linolenic acid by two steps: conversion of C18:3 ω-3 to C20:5 ω-3 (eicosapentaenoic acid) then to C22:5 ω-3 (docosapentaenoic acid) and finally to C24:5 ω-3 (tetracosapentaenoic acid) in the endoplasmic reticulum. The second step consists of a single-ring peroxisomal β-oxidation of C24:6 ω-3 to C22:6 ω-3 (DHA) [13]. This step of peroxisomal β-oxidation requires the intervention of straight-chain acyl-CoA oxidase (SCOX), D-bifunctional protein (DBP), 3-ketoacyl-CoA thiolase, and sterol carrier protein (SCPx).

## 3. Impact of DHA on Cell Death and Oxidative Stress Induced in Microglial: In Vitro Studies

In the pathophysiology of neurodegenerative diseases, a process of cell death is described that is most often accompanied by oxidative stress. This cell death process mainly affects neurons, but other cells of the nervous system, such as oligodendrocytes or microglia, can also be affected. This raises the question of whether DHA can prevent microglia cell death. Indeed, few studies have targeted the action of DHA on microglial cell death. Among the studies carried out, it was shown that DHA could protect microglial cells from death induced by oxidized derivatives of cholesterol and oxysterols, identified in the cerebrospinal fluid, plasma, and tissue of patients with different neurodegenerative diseases (multiple sclerosis (MS), Alzheimer’s disease (AD), X-linked adrenoleukodystrophy). In this study, 7-ketocholesterol induces cell death characterized by an apoptotic process associated with oxidative stress but also with autophagy in a microglial murine cell line, the BV-2 line. This process is called oxiapoptophagy (OXIdative stress + APOPTOsis + autoPHAGY) [14]. DHA used at 12 µM is capable of inhibiting the oxiapoptophagy process [15]. Another study has shown that at too high concentrations, DHA can, on the contrary, induce cell death. In a BV-2 cell model, DHA used at 200 µM is capable of inducing pyroptosis [16]. This pyroptosis process can be inhibited by 12-lipoxygenase (12-LOX, Alox12e) inhibitors [17].

Concerning oxidative stress, a team showed that DHA was able to inhibit oxidative stress and increase antioxidant defenses in microglial cells. The use of oligomeric amyloid-β peptide (oAβ) induces oxidative stress via the production of ROS in primary mouse microglia and in immortalized mouse microglia: the BV-2 line. This oxidative stress can be inhibited by the use of DHA (10 µM), which can also positively regulate antioxidant pathways by involving the nuclear factor (erythroid-derived 2)-like 2/heme oxygenase-1 (Nrf2/HO-1) [18]. In another model where BV-2 cells were stimulated with lipopolysaccharide (LPS), it was shown that DHA was indeed able to decrease oxidative stress production and increase antioxidant responses via Nrf2/HO-1 [19]. In this model, the use of quercetin coupled with DHA increases the beneficial effects of these two molecules and reduces the concentrations of DHA (IC50 in the range of 60-80 µM used alone) necessary to observe a positive effect. The most effective combination seems to be the following: quercetin (2.5 µM) in combination with DHA (10 µM) [20].

Few data are available on the effect of DHA on cell death of microglia or oxidative stress; the main investigations concern the relationship between microglia and inflammation, which will be developed in the following paragraph. The different results presented are summarized in Table 1.

## 4. Impact of DHA on Inflammation Induced by Microglia in Neurodegenerative Diseases: In Vitro and Animal Studies

Immune activation of the central nervous system is present in neurodegenerative pathologies and involves microglia, major players in neuroinflammation [33]. This inflammatory response, necessary to respond to aggression, can lead to the production of neurotoxic factors when prolonged. It is also characteristic of many neurodegenerative diseases, such as AD, Parkinson’s disease (PD), amyotrophic lateral sclerosis (ALS), and MS [3]. For several years, the anti-inflammatory capacity of DHA or derivatives forms of DHA has been demonstrated.

In the first part, we present the results obtained in vitro (summarized in Table 1) and in the second part, we present the data obtained in vivo with neurodegenerative models (summarized in Table 2).

### 4.1. In Vitro Results

Using the BV-2 microglial cell line, Lu et al. have shown that DHA (30 µM) reduced expression of inducible nitric oxide synthase (iNOS), cyclooxygenase-2 (COX-2), and tumor necrosis factor α (TNF-α) induced by interferon-γ (INF-γ), and antagonized IFN-γ induced NO production [21]. Inoue et al. investigated the implication of the sirtuin (SIRT) signaling in the anti-inflammatory response mediated by microglia, with BV-2 cells and with the MG6 line. They also found that DHA inhibited production of TNF-α and IL-6 induced by stimulation in two cellular models (BV-2 cells and MG6 microglia), but had no effect on IL-10 production induced by LPS. Results obtained with the MG6 microglia cells and a treatment with DHA (100 µM) + eicopentaenoic acid (EPA, 100 µM) suggest that the anti-inflammatory properties of DHA and/or EPA could be due to a SIRT1-mediated NF-κB (nuclear factor-kappa B) p65 deacetylation, through a positive feedback regulation of SIRT1 gene expression [22]. Others studies have shown that DHA (30 µM) decreased IL-1β [17,23] and IL-6 [23] expression in BV-2 cells stimulated with LPS. In primary cultures of mice microglial cells, treatment by DHA (20 µM to 80 µM) prior to LPS induction significantly attenuated LPS-induced NO and TNF-α release in a dose dependent manner. The inhibitory effect of DHA (20 µM) on TNF-α and NO release was also observed when cells were treated with myelin + IFNγ. In the same study, the authors observed that DHA probably modulates phenotypic polarization of microglia, with upregulation of M2-associated genes (including chemokine ligands (*CCL), CCL2, CCL17, Arg1*, and *IL-5*) and downregulation of M1-associated genes (including *IL-6, CCL5, TNF-α*, and *IL-1α*) [24]. With the human CHME3 microglial cells treated by DHA (0.1 to 1 µM) and exposed to amyloid-β42 (Aβ42), Hjorth et al. have shown a decrease in the levels of TNF-α and cluster of differentiation (CD) CD40 and CD86, as well as an increase in CD206 [25].

Furthermore, in rat glial primary cell cultures, DHA (100 µM) seems to have an active role in the regulation of the pro-inflammatory response. Indeed, pre-incubation of rat glial primary cell cultures with DHA before LPS/IFN-γ stimulation led to a decrease in the DNA binding activity of the activating protein-1 (AP1) and phosphorylation of c-Jun N-terminal kinase (JNK) and c-Jun. This pre-incubation also led to an increase of the expression of Nrf2 and HO-1. Using DHA before IFN-γ stimulation counteracted the elevation of the pro-inflammatory cytokines TNF-α, IL-1β, IL-6, CCL2, and C-C chemokine receptor type 2 (CCR2) [26]. With macrophages, Cai et al. have demonstrated that 24 h of DHA (20 µM) treatment increased the expression of arginase-1 and TGF-β and suppressed production of CCL2, C-X-C motif chemokine ligand 10 (CXCL10), IL-1α, and TNF-α in primary macrophage cultures [43]. Reduction of the production of cytokines TNF-α and IL-6 could be induced through toll-like receptor-3 (TLR-3) and TLR-4 activation in EOC20 microglia cells treated by polyinosinic–polycytidylic acid (synthetic double-stranded RNA consisting of one strand of poly(inosinic acid) and one strand of poly(cytidyl acid) paired by wobble pairing, structurally similar to the double-stranded RNA of certain viruses, triggering an immune response) or 10 μg/mL of imiquimod (immune response modifier) [27].

Other forms of DHA have been used to decrease LPS-induced inflammation in BV-2 cells. Triglycerides forms of DHA (20 µM), or endogenous derivatives, have the capability to significantly reduce the production of IL-6 and TNF-α [29]. N-docosahexaenoyl dopamine (DHDA, 2 µM) decreases production of IL-6 and CCL-20 (macrophage-inflammatory protein-3α). Authors have also demonstrated that the level of prostaglandine E2 is reduced by using DHDA [30]. Synaptamide, an endogenous metabolite of DHA, leads to similar effects on inflammation. Using primary cultures of rat microglia and BV-2 cells, Park et al. found that synaptamide suppressed LPS-induced TNF-α and iNOS mRNA expression in a dose dependent manner. Furthermore, synaptamide decreased expression of IL-1β, IL-6, and CCL2. The authors suggest that the anti-inflammatory effects of synaptamide could be due to its fixation on the GPR110 receptor, as the synaptamide effects were suppressed by blocking synaptamide binding to it. This interaction could lead to an upregulation of cyclic adenosine 3′,5′-monophosphate/protein kinase A (cAMP/PKA) signaling by inhibiting NF-κB p65 nuclear translocation [31,32]. Pro-inflammatory effects have also been demonstrated with resolvins (RvD) that are metabolites of DHA. RvD2 could counteract the mRNA pro-inflammatory upregulation induced by LPS (CD11b, ionized calcium binding adaptor molecule 1 (Iba-1), TNF-α, NF-κB p65, iNOS, IL-1, IL-18, IL-6, the nuclear factor of kappa light polypeptide gene enhancer in the B-cells inhibitor, alpha (IkBα), the the inhibitor of the nuclear factor kappa-B kinase subunit β (IKKβ), and IL-1β) and decrease the ROS production [44].

In their recent study, Chang et al. showed that the neuroprotective and anti-inflammatory properties of DHA could attenuate effects of Japanese encephalitis virus (JEV). This virus, when it invades the central nervous system, causes a robust inflammatory response that leads to neuronal cell death. When infected with the JEV, primary neuron/glia rat primary cultures (i.e., neurons, astrocytes, and microglial cells), the authors measured an increase of neurotoxin cytokines production (NO, TNF-α, IL-1 β, prostaglandine E2 (PGE2), and ROS) that is counteracted by a DHA treatment (25 or 50 µM, 12 h) [28].

Taken together, these studies demonstrate the ability of DHA or its derivatives to limit the inflammatory effects, to be neuroprotective, and even to promote anti-viral effects in different types of cell cultures and in different models of inflammation.

Some data obtained in vitro are also observed in in vivo models.

### 4.2. In Vivo Models

#### 4.2.1. Neuroinflammation Mediated by LPS

A neuroinflammation model could be obtained by injection of LPS intraperitoneally (i.p. injection) or directly in the brain of C57BL/6J mice; i.p. injection of LPS works to increase the levels of TNF-α, IL-1β, IL-6, iNOS, and CCL2 in the brain. Expression of these mRNA and the number of Iba-1 positive cells significantly decrease when synaptamide is administered (5 mg/kg). These effects are not observed when synaptamide is injected in G-protein-coupled receptor 110 (GPR110) knock-out mice after LPS induction, suggesting that anti-inflammatory effects in vivo depend on GPR110 [32]. In another study, fish hydrolysate supplementation, containing low amounts of DHA (143 µg in 150 µL of fish hydrolysate supplement/day), reduced expression of TLR4, cytokines (IL-6, TNF-α, IL-1β,), CCL2, and the inhibitor of the nuclear factor κB (IκB) in mice with LPS induction. DHA alone reduced only IL-6 expression. Furthermore, the authors observed lower expressions of CD86, CD68, and CD11b M1-markers in DHA groups compared to LPS groups fed with fish hydrolysate, but no effect on CD206, CD36, and Arg1-M2 markers were observed in the hippocampus. The authors suggest that the DHA effect is potentialized in fish oil by other low molecular weight peptides [34]. As well as in in vitro experiment, RvD2 could inhibit microglia activation in PD model LPS-treated animals (RvD2 per 25, 50, and 100 ng/kg, 27 days post LPS-treatment). The activation of microglia is significantly lower after treating animals with RvD2 (25, 50, and 100 ng/kg, 27 days post LPS-treatment) than in the vehicle group (more ramified microglia and less CD11b content of the substantia nigra pars compacta in the treated group). Furthermore, IL-18, IL-6, NO, TNF-α, and IL-1β production induced by LPS were significantly inhibited by RvD2 in a dose dependent manner (2,5 µM to 20 µM), in vivo (serum) and in vitro, probably through inhibition of the TLR4/NF-kB pathway [44]. In their study, Fourrier et al. showed that 1-palmitoyl,2-docosahexaenoyl-PC (PC-DHA, 4.33 µg/g of mouse) attenuates the effect of LPS in mice brains when injected 24 h prior to LPS induction. Particularly, IL-6 production induced by LPS significantly decreased in the hippocampus. The in vitro results on BV-2 microglial cells suggest that these effects could be mediated by GP130 receptors [23]. Some authors did not observe significant effects of DHA dietary supplementation in vivo, even with a DHA increase in the brain. In [35], even though supplementing piglets with herring oil (DHA: 32.30% W/W total fatty acid), starting at postnatal day 2, led to a concentration increase of 27% at 14 postnatal days compared to piglets without supplementation, no attenuation of the LPS induced inflammation was observed. In a different model of LPS-induced inflammation (intracerebroventricular LPS injections in C57Bl/6 male mice), similar results were obtained. Indeed, DHA (fish oil, 1.4% of total fatty acids) increased more than 90% in fat-1 mice compared to their wildtype littermates, and a similar increase was observed in the brains of fish oil-fed mice compared to wildtype animals fed with a safflower diet. Despite this increase, no change in the expression of pro-inflammatory genes was found [36]. These contradictory results could be explained by the different modalities of LPS injection, the different animal models used, and the variations in analysis time.

#### 4.2.2. Alzheimer Disease

As a model for Alzheimer disease, different transgenic kinds of mice have been studied to observe effects of DHA or its derivatives. In a triple-transgenic mouse model of Alzheimer disease, neuroprotection D1 (NPD1) (50 nM) downregulates Aβ42-induced expression of COX-2, TNF-α, and B-94, a TNF-α-inducible pro-inflammatory element [37]. Increasing brain DHA level after intracerebroventricular amyloid-β infusion led to a decrease in Iba-1 microglial cells counted in fat-1 mice and lower degenerative neurons in mice supplemented with fish oil containing 1.5% DHA (among other fatty acids). Authors also observed significantly higher branching complexity (CA1, CA3, and dentate gyrus) in fat-1 and wild type mice fed with fish oil than in wild type mice fed with safflower oil. These results suggest that dietary supplement in fatty acids, including DHA decreased microglial responses to amyloid-β infusion [38]. In another model of AD mice, induced by co-administration of D-galactose and aluminium chloride, supplementation with DHA (200 mg/kg) + EGb761 (*Ginkgo biloba* standardized extract) enhanced behavioral recovery and protein phosphatase 2A expression, a major protein phosphatase of tau in the brain, while it downregulated TNF-α expression (both in the hippocampus, CA3) [39]. Sharman et al. tried a different combination of supplements instead of using DHA, ALA (α-lipoic acid), epigallocatechin-3-gallate, or curcumin alone. Reductions in amyloid plaque load, microglial activation, Aβ40/Aβ42 levels, and memory deficits in male Tg2576 mice (a familial AD model) have been observed by using a combination of EGCG, DHA (50 mg/kg body weight), and ALA [41].

Surprisingly, TgCRND8 mice (transgenic mice model for AD) supplemented with 0.246% of DHA (wt/wt) in a whole-food diet, exhibited higher TNF-α expression compared with control groups, corresponding with observed behavioral impairment [40].

#### 4.2.3. Multiple Sclerosis

Multiple sclerosis (MS) is an immune pathology leading to demyelination and dramatic alteration of central and peripheral nervous systems.

The cuprizone mouse presents a massive demyelination and is a validate model of MS. When cuprizone mice were fed with DHA + EPA (15 g/kg for 5 weeks), myelin integrity was improved, and behavioral deficits were reduced (better scores on the Morris water maze test and with the rotarod test). The authors measured iNOS and CD16 expression, as well as CD206, YM1/2, and Arg1. They found that supplementation with n-3 PUFA suppressed the increase of M1-associated genes but increased the expression of M2-related genes [24]. Experimental autoimmune encephalomyelitis (EAE) is another commonly used model for MS. Mancera et al. have shown that feeding EAE mice with DHA (250 mg/kg/day, 15 days before EAE induction and 41 days after EAE induction) with the triglyceride form of the omega-3 polyunsaturated fatty acid docosahexaenoic acid (TG-DHA) significantly improved the clinical score in a dose and time dependent manner, along with weight profile [29]. A recent study concluded that EAE onset and severity were reduced when mice were fed with a DHA diet (phospholipid-DHA, 0.3% or 1%, and triacylglycerol-DHA, 0.3%), compared to mice fed with low α-linolenic acid [42].

### 4.3. Some Other Neuropathologies

Beneficial effects of DHA or derivatives have been observed in other models of neuroinflammation. Even if these pathologies are not neurodegenerative diseases, they are of interest because of their neuroinflammation component accompanied by microglial activation and pro-inflammatory factor release.

For example, synaptamide could be used to treat chronic neuropathic pain. In vitro, the addition of synaptamide to the SIM-A9 murine microglia cell line prevented LPS-induced NO overproduction and ROS production. In vivo, rats with sciatic nerve chronic constriction injury (CCI) treated with synaptamide showed lower concentrations of hippocampal Iba-1, CD86, IL-6, and IL-1β than the CCI group without synaptamide treatment. Furthermore, more doublecortin-positive neurons and proliferating cell nuclear-positive cells have been counted in the dentate gyrus subgranular zone in the CCI synaptamide treated rats compared to the CCI rats. Behavioral improvements were also observed in the synaptamide-treated groups [45].

Others have shown anti-inflammatory effects of NPD-1 in old mice in a model of postoperative delirium, with reduction of IL-6, TNF-α, glial fibrillary acidic protein (GFAP), and Iba-1 compared with controls. IL-10 increase was also observed [46].

Taken together, all of these studies suggest strong benefits of DHA mediated by microglial cells on neuroinflammation in neurodegenerative disorders. However, the mechanisms of action of the anti-inflammatory in vivo are still not elucidated.

## 5. DHA, Clinical Trials, and Neurodegenerative Diseases

Various results of clinical trials have been published concerning the use of DHA in neurodegenerative diseases (summarized in Table 3). A majority of clinical trials focus on AD. In a first clinical trial on this pathology, a food supplementation composed of xanthophyll carotenoids and omega 3 fatty acids was tested [47]. Two conditions were tested: the first condition was lutein/meso-zeaxanthin/zeaxanthin at 10:10:2 mg/day and the second condition was the formulation used in the first condition plus 1 g/day of fish oil containing 430 mg DHA and 90 mg EPA. It turned out that the formulation containing DHA was the most effective in slowing down AD, showing that the consumption of xanthophyll carotenoids combined with DHA (fish oil) has a better protective effect than xanthophyll carotenoids used alone [47]. Disease progression is reduced with this formulation with improvement in memory, sight, and mood. An OmegAD clinical trial (NCT00211159), enrolling 204 participants, studied the effects of DHA-rich dietary supplementation on cognitive impairment in patients with AD. Preliminary results showed that DHA (capsule EPAX 1050TG; Pronova Biocare A/S, one capsule: 430 mg DHA and 150 mg EPA) supplementation for 6 months induces DNA hypomethylation in blood cells [48].These results provide a new possible mechanism of action for these compounds: they could modulate gene expression by hypomethylation. The authors postulate that it could then be interesting to treat AD with hypomethylating agents [48]. Another work from the OmegAD clinical trial (NCT00211159) studied the plasma levels of fatty acids following DHA intake. It was shown that the higher the plasma levels of omega-3 fatty acids, the better the cognitive functioning, regardless of gender [49]. However, body weight is important and DHA doses should be adjusted to it [49]. Another part of the clinical trial was to study immune function. Since it is difficult to work on microglia directly, the authors used peripheral blood mononuclear cells (PBMCs), which can infiltrate the brains of Alzheimer’s patients like T lymphocytes and monocytes and participate in the development of inflammation. PBMCs were recovered before and after supplementation with DHA and EPA and then treated with the Aβ40 peptide. DHA/EPA supplementation prevented the reduction of specialized proresolving mediators (SPM, lipoxin A4, and RvD1 released from PBMCs) [50]. Furthermore, inflammation resolution is disrupted in patients with AD; DHA/EPA supplementation (EPAX1050TG; Pronova Biocare A/S, Lysaker, Norway) could improve it [50]. As the authors point out, it remains to be determined whether the same effects can be observed in microglia and whether the use of SPM or their precursors could be effective in the treatment of AD [50]. Another component of the study was to evaluate the effects of oral dietary omega-3 supplementation on inflammatory biomarkers and oxidative stress. Patients were supplemented for 6 months with a DHA/EPA complex (four capsules of EPAX 1050TG, i.e., 1.7 g of DHA and 0.6 g of EPA); urine samples were collected before and after supplementation [51]. In these samples, the levels of major F2-isoprostane, 8-iso-prostaglandinF2α (biomarker oxidative stress), and 15-keto-dihydro-prostaglandin F2α (biomarker of inflammation) were measured [51]. The results obtained indicate that DHA/EPA supplementation does not have a well-defined effect on oxidative stress as measured but may have a possible role in immunoregulation. Since AD affects the brain, it was interesting to know if fatty acids are able to cross the blood–brain barrier. Therefore, fatty acid profiling was performed in cerebrospinal fluid (CSF) to assess whether supplementation was able to alter this profile [52]. Patients received 6 months of DHA supplementation (four capsules of EPAX 1050TG, i.e., 1.7 g of DHA and 0.6 g of EPA). After 6 months, changes in the fatty acid profile were observed, with a significant increase in eicosapentaenoic acid (EPA), DHA, and total n-3 FA levels in CSF [52]. A correlation was also made with the markers of AD and, the more DHA levels increased in the CSF, the more changes there were in the biomarkers of the pathology (tau, phosphorylation of the tau protein, IL-1 receptor). Their results also showed that supplementation failed to stop disease progression and that DHA supplementation would likely need to be taken early to see an effect on disease progression [52]. In AD, deposits of the Aβ protein are present in the brain. Transthyretin (TTR) can bind to amyloid β and thus reduce its presence. Patients received DHA/EPA supplementation (four capsules of EPAX 1050TG, i.e., 1.7 g of DHA and 0.6 g of EPA) for 6 months; it was observed that this treatment could increase plasma levels of TTR, which could influence Aβ peptide deposits in the brain, results that need to be confirmed by further experiments [53]. DHA/EPA supplementation was also evaluated on gene expression in peripheral blood mononuclear cells [54]. Patients received a DHA/EPA complex (four capsules of EPAX 1050TG, i.e., 1.7 g of DHA and 0.6 g of EPA) for 6 months and the expression of 8000 genes was studied. Modulations of the expression of several genes (decrease (10) or increase (9)) were measured. The upregulated genes are *MS4A3* (role in signal transduction), *NAIP* (apoptosis inhibitory protein), *DRG1* (stress and hormone responses, cell growth, differentiation), *CD36* (cell adhesion, cell migration), *HSD17B11* (regulation of inflammation, modulation of intracellular glucocorticoid levels), *RAB27A* (signal transduction), *CASP4* (inflammatory caspase), *SUPT4H1* (RNA synthesis), and *UBE2V1* (ubiquitination). The negatively regulated genes are *RHOB* (inflammation), *VCP* (vesicle transport, fusion, and ubiquitin-dependent protein degradation), *LOC3999491*, *ZNF24* (transcription factor), *SORL1* (regulation of processing of amyloid precursor protein), *MAN2A1* (inflammation regulation), *PARP1* (differentiation, proliferation, tumor transformation, DNA damage reparation), *SSRP1* (action on transcription), *ARIH1* (ubiquitination process), and *ANAPC5* (cell cycle progression). Many of these genes are involved in inflammation regulation and neurodegeneration, and in ubiquitination processes [54]. The impact of DHA (four capsules of EPAX 1050TG, i.e., 1.7 g of DHA and 0.6 g of EPA) on inflammation was confirmed by another study performed in this clinical trial, which showed that DHA decreased the release of PGF2α from LPS-stimulated PBMCs and that it could be hypothesized that DHA could act via anti-inflammatory and neuroprotective lipid mediators on the resolution phase of inflammation [55]. Using the same protocol (PBMCs treated with LPS following DHA/EPA supplementation, four capsules of EPAX 1050TG, i.e., 1.7 g of DHA and 0.6 g of EPA), the authors also showed that the increase in plasma DHA concentration was correlated with a reduction in the release of IL-1β, IL-6, and the granulocyte colony-stimulating factor of PBMCs [56].

In an independent study of the OmegAD study, omega-3 fatty acid supplementation (capsules containing a total of 625 mg of DHA and 600 mg of EPA) increased plasma DHA and EPA concentrations in people with cognitive impairment no dementia, and AD. However, no beneficial effect on cognition and mood was observed in these populations. As emphasised by the authors, the sample used to conduct this study was quite small (76 participants), and the duration of the study was short (4 months). One hypothesis raised by the authors was to adapt the dose according to the pathology studied and the level of progression of the pathology [57]. These same observations were made in a study carried out by Paul S. Aisen’s group, where DHA supplementation did not slow cognitive decline in patients with mild or moderate AD [58]. This study involved 402 patients and lasted for 18 months. DHA supplementation was performed with an algal-derived DHA (Martek Biosciences, Columbia, Maryland) in capsule form. Twice a day, patients took 1 g, for a total daily dose of 2 g, knowing that these capsules contained approximately 45% to 55% DHA by weight and did not contain eicosapentaenoic acid. The authors suggest that DHA may have an effect if the patients do not have overt dementia [58]. A third study shows that omega-3 supplementation (EPAX1050TG™ from Pro-nova Biocare A/S, Lysaker, Norway, four capsules) in patients with mild to moderate AD did not induce effects on neuropsychiatric symptoms, but had possible positive effects on depressive symptoms in non-ApoEω4 carriers and on agitation symptoms in ApoEω4 carriers [59]. In contrast to these three studies, a supplementation study using Aravita capsules (Suntory Ltd., Osaka, Japan), containing 40 mg/capsule of arachidonic acid (ARA) and DHA and 0.16 mg/capsule of astaxanthin (antioxidant of PUFA), showed significant improvements in the memory of patients with organic brain damage or mild cognitive impairment [60]. The authors hypothesize that these changes may be due to neural circuit remodeling (possible upregulation of synaptogenesis and/or neurogenesis with ARA), as well as improvement of membrane function and regional cerebral blood flow by DHA [60].

A few clinical trials have focused on spinocerebellar ataxias, which are very heterogeneous neurodegenerative diseases, clinically and genetically. The main characteristic of these pathologies is cerebellar syndrome, associated with walking and balance disorders. Spinocerebellar ataxia 38 (SCA38) is caused by a mutation in the elongation of the very long chain fatty acid protein 5 (ELOVL5) gene and is associated with reduced serum DHA levels. The team of Borroni et al. studied the effect of short-term (16–40 weeks) and long-term (2 years) DHA supplementation [61,62]. The DHA was derived from algal oil (Sofedus, Milan, Italy) and administered as sachets dosed at 600 mg/day. Improvement in clinical symptoms and no degradation of neurophysiological parameters were observed following fish oil-derived DHA intake.

The published results of clinical studies (mainly on AD) underline the interest in DHA, particularly through its ability to cross the blood–brain barrier and to influence inflammation. It is not known to what extent DHA crosses this barrier and whether microglia, the orchestral leader of inflammatory reactions in the brain, is affected by DHA. Many authors also mention the need to adapt the dose according to the severity of the disease or the weight of the patients. With DHA, the problem is not that it does not cross the blood–brain barrier, but that the quantity of DHA that crosses the barrier is not sufficient and does not only target the microglia. It is therefore important to look at the contribution of nanotechnologies in the targeting of therapeutic molecules of interest, such as DHA, to the microglia.

## 6. Brain Nanomedicine and Microglia

The DHA used in clinical studies comes from either oil, algae, or industry. In the OmegAD study, the DHA came from Pronova Biocare. This company uses a process to deodorize its EPAX triglyceride oils, inducing neither taste nor odor. They use enzymes to form the triglycerides. Their product can be in the form of chewable capsules or a liquid formula. In these different possibilities, DHA is not targeted to a cerebral distribution. DHA is delivered systemically, and authors have observed changes at the plasma, cerebrospinal fluid, and cognitive levels [49,52]. It might be interesting to look at targeting DHA to the brain by using nanotechnology-based drug delivery systems to counteract the low penetration efficiency of drugs to the central nervous system due to the presence of the BBB. Two possibilities exist to reach the brain: either a direct passage that will damage the BBB, or an indirect passage via nanomedicines targeting the brain either orally or by nasal delivery through a spray. The results presented in this section will focus on those obtained on microglia, whether from primary culture, lineages, or animal models, in the context of neurodegenerative diseases.

### 6.1. Nanoparticles

For this purpose, nanoparticles (size ranging from 1 to 100 nm) have been developed, which are 3D encapsulation systems that allow the transport of a molecule, and which can be functionalized by ligands, antibodies, or other molecules for targeting toward a target organ. These nanoparticles can be made from natural or synthetic polymers, metals, or from lipids.

#### 6.1.1. Lipid-Based Nanoparticles

**Figure 3:**
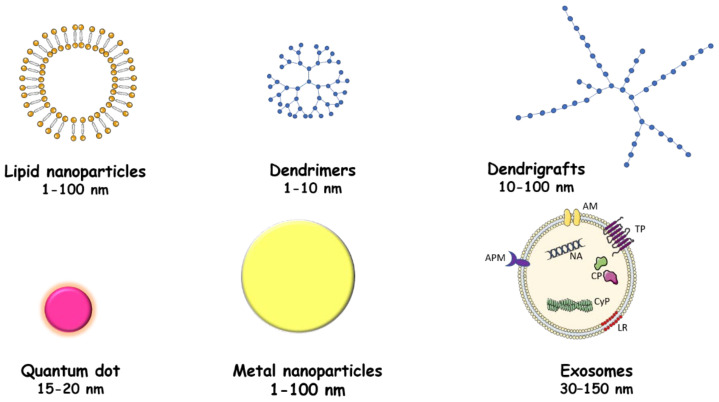
Representation of different delivery systems for therapeutic molecules. Different systems are used to deliver therapeutic molecules: lipidic nanoparticles, dendrimers and dendrigrafts, quantum dots and metallic nanoparticles, and exosomes. AM: adhesion molecules; TP: transmembrane proteins; APM: antigen presenting molecules; NA: nucleic acid; CP: cytosolic proteins; CyP: cytoskeletal proteins; LR: lipid rafts.

These lipid-based nanoparticles have the advantages of being non-toxic and having a high loading capacity for hydrophilic or non-hydrophilic molecules of interest, as well as a capacity to get through the BBB (Figure 3).

Nanoparticles have been formed from DHA and its hydroxylated derivative (DHAH); these are directly active without the need to load other molecules. These nanoparticles were tested on primary cultures of microglia obtained from rats. The viability of the cells is not affected by the use of these DHA/DHAH nanoparticles; however, an anti-inflammatory action of these nanoparticles is noted when the microglial cells are stimulated by LPS, with a decrease in the release of TNFα, IL-6, and IL-1β [63]. This work allowed us to show in vitro that these DHA/DHAH nanoparticles were not toxic and could reduce the release of proinflammatory cytokines by microglial cells [63]. This approach must still be validated in vivo in animals before testing in humans. This same team had already used lipid nanoparticles coated with chitosan, with the surface modified with a transactivator of transcription (TAT) peptide and loaded with GDNF (glial cell-derived neurotrophic factor) (CS-NLC-TAT-GDNF) [64]. In a 1-methyl-4-phenyl-1,2,3,6-tetrahydropyridine (MPTP)-induced Parkinson’s mouse model, intranasal administration of CS-NLC-TAT-GDNF led to modulation of microglial activation [64]. The use of lipid nanoparticles can induce toxicity and induce activation of brain microglia (involvement of the P2X7/caspase-1/IL-1β pathway). It has been shown that by modifying these lipid nanoparticles with PEGylation, this microglial activation could be reduced [65]. Nanoparticles can pass through the nasal epithelium and reach the brain by two different routes: (a) the extracellular route, the most common mechanism of delivery of therapeutics to the brain, via passive transport through the nasal epithelium, and (b) the intracellular route, involving endocytosis in the branches of the olfactory and trigeminal nerves followed by axonal transport in the brain [66].

Nanoparticles were synthesized from high-density lipoprotein (HDL) associated with apolipoprotein E [67,68]. These nanoparticles are able to enter cerebral vessels and accumulate around Aβ aggregates. First, ApoE-associated nanoparticles were shown to reduce Aβ deposition, attenuate microgliosis, improve neurological changes, and reduce memory deficits in an animal model of AD [67]. In a second step, α-Mangostin (α-M), a polyphenolic agent capable of inhibiting the formation of Aβ oligomers and fibrils and accelerating the cellular degradation of Aβ, was added to these nanoparticles [68]. These α-M-loaded nanoparticles are able to promote the uptake and degradation of Aβ1-42 by microglia more than unloaded nanoparticles [68]. This same system of reconstituted and modified HDL was used with monosialotetrahexosylganglioside (GM1), possessing high Aβ binding affinity [69]. This nanosystem promotes Aβ degradation by microglia following intranasal administration. This structure was used to load a neuroprotective peptide NAP, αNAP-GM1-rHDL, which was able to reduce Aβ deposition more efficiently than the nanostructure alone or α-NAP alone, in mouse models of AD after intranasal administration [69]. 

Oridonin, a natural diterpenoid compound isolated from the Chinese herb *Rabdosia rubescens*, was loaded into commercial lipid nanocarriers, Lipofundin^®^ (MCT, 10% for infusion, B. Braun AG, Melsungen, Germany), and then given orally or injected into mice constituting an animal model of cerebral amyloidosis for AD, transgenic APP/PS1 mice. Regardless of the mode of injection, oridonin-loaded nanoparticles were able to attenuate microglia activation [66]. In an in vitro model of microglia (line N9) stimulated by LPS, these nanoparticles are able to inhibit the inflammatory response by reducing the NO concentration and decreasing mRNA expression of iNOS, IL-1β, and IL-6 [70].

Nanoparticles with a lipid core (capric/caprylic triglycerides) were loaded with indomethacin (a non-steroidal, anti-inflammatory drug) and their impacts on neuroinflammation were evaluated on organotypic rat hippocampal cultures after treatment with Aβ1-42 peptide, mimicking AD [71]. The use of these nanoparticles allows for the decrease of the TNF-α and the increase of IL-6 induced by Aβ1-42, but also the increase of the release of interleukin-10 [68]. The activation of microglia is reduced by the use of these nanoparticles [71]. 

The use of lipid nanoparticles seems to be a promising way to deliver molecules of interest to the brain by targeting microglia, while controlling their possible toxicity via surface modifications.

#### 6.1.2. Metal Nanoparticles

Different types of metals can be used to create nanoparticles, such as titanium, gold, or silver (Figure 3).

Regarding titanium nanoparticles, in vitro experiments on BV-2 microglia cells showed that titanium dioxide nanotubes, which were functionalized with 3-aminopropyl triethoxysilane (APTES), allowing the addition of amine functions for drug molecule conjugation, do not induce toxicity or activation of these cells [72]. Titanium dioxide nanoparticles are able to move in the brain by decreasing the transendothelial electrical resistance and by disrupting the tight junctions between the endothelial cells of the brain capillaries [73]. Titanium dioxide (TiO_2_) nanoparticles are able, without being functionalized by other molecules, to drive microglia toward the proinflammatory activation phenotype [74]. This change of phenotype would be specific to microglia, as astrocytes do not change their phenotype following the use of these nanoparticles [74].

Gold nanoparticles have also been developed. Some have been tested on primary cultures of microglia. They consist of 18 atoms of gold and are stabilized with glutathione ligands. The authors of this study showed that these Au18 gold nanoclusters (NCs) have, at low concentrations, anti-inflammatory signaling (reduction of (IL1-β) levels, unchanged levels of TNF-α or Ym1/2) but, at higher concentrations, they can have pro-inflammatory activity [75]. The authors suggested that the presence of glutathione could be the source of this anti-inflammatory activity. Gold nanoparticles (AuNCs) were functionalized with dihydrolipoic acid (DHLA-AuNCs), a neuroprotective antioxidant. The BV2 microglial line was used to evaluate their effects on microglial polarization. These nanoparticles induced a polarization toward the M2-like phenotype as well as a decrease of oxidative stress, a reduction of NF-kB signaling, and an increase of cell survival (increase of autophagy, inhibition, of apoptosis) [76]. Microglial changes also impacted neuronal cells by improving neurogenesis and reducing astrogliosis [76]. Another team used the root extract of *Paeonia moutan*, woody trees which are used in traditional Chinese medicine, to functionalize gold nanoparticles. Still, on a BV2 cell model, it was shown that these nanoparticles were able to decrease oxidative stress and inhibit the synthesis of pro-inflammatory cytokines following stimulation by LPS [73]. The effect of these nanoparticles on oxidative stress and inflammation was found in a model of parkinsonian mice, associated with an improvement in motor disorders [77]. An extract of *Ephedra sinica* Stapf was used to functionalize gold nanoparticles. These nanoparticles were able to decrease the production of pro-inflammatory mediators and cytokines (TNF-α, IL-1β, and IL-6) following primary microglia and BV-2 microglial cells induction by LPS (decrease of IκB kinase-α/β, NF-κB, Janus-activated kinase/signal transducers and activators of transcription, mitogen-activated protein kinase, and phospholipase D signaling pathways) [78]. Gold nanoparticles were synthesized with quercetin and then used on BV2 cells stimulated with LPS. The release of pro-inflammatory prostaglandin, prostaglandin E2, NO, upregulation of COX, inducible NO synthase mRNA, and protein levels were strongly inhibited by gold/quercetin nanoparticles [79]. The effects of these nanoparticles are superior to the use of quercetin alone. The use of these gold-quercetin nanoparticles could thus decrease the activation of microglia. A gold cluster with a positively charged tridecapeptide Sv (Au25Sv9) (peptide Sv: (H2N-CCYGGPKKKRKVG-COOH)) was produced, and it was shown that these particles were able to attenuate the cytotoxicity of stimulated microglia cells toward neuronal cells [80]. These nanoparticles inhibited IL-6, TNF-α, and NO secretions by suppressing the activation of NF-κB and p38 pathways. The action of these nanoparticles was also observed directly on neuronal cells, indicating that these nanoparticles could target microglia and neurons and could be an effective therapeutic approach [80].

Complex nanoparticles combining a metallic part and a polymer were produced in the following way: diblock polymer Man-PCB-PB was synthesized and then assembled to form nanoparticles to enclose the hydrophobic part fingolimod and zinc [81]. These nanoparticles were named Man-PCB-PB/ZnO/fingolimod NPs or MCPZF NPs. They were then linked to a signal transducer and activator of transcription 3 small interfering RNA (siSTAT3) to give Man-PCB-PB/ZnO/fingolimod/siSTAT3 NPs or MCPZFS NPs [81]. These nanoparticles promote phagocytosis of Aβ by microglia and decrease the release of pro-inflammatory cytokines [81].

Magnetic iron oxide (maghemite, γ-Fe2O3) nanoparticles (high surface-to-volume ratio, diameter 21 ± 3.5 nm, magnetic, biocompatible, relatively non-toxic, biodegradable) were used to deliver the following peptide: fibrin γ377–395 peptide [82]. The effect of these nanoparticles is different depending on the age and the state of AD. Indeed, in the early stages, the reduction of microglial cell activation following the action of these nanoparticles increases the number of neurons with hyperphosphorylated tau in transgenic mice [82]. Abnormal hyperphosphorylation of tau protein in sites that are not normally phosphorylated leads to the formation of neurofibrillary tangles (NFTs) in neuronal cell bodies and sometimes in glial cells. Hyperphosphorylation and NFT formation induce an inability for tau protein to bind to microtubules, resulting in alterations in axonal trafficking leading to changes in neuronal function and viability. These processes participate in synaptic dysfunction and neurodegeneration. On the other hand, in older mice, the reduction of microglial cell activation reduces the severity of tau pathology [82]. The number of neurons with hyperphosphorylated tau and the number of neurons with tangles are reduced in animals receiving the γ377–395 fibrin peptide-nanoparticle conjugate compared with control animals [82].

#### 6.1.3. Polymer Nanoparticles and Mesoporous Silica Nanoparticles

Nanoparticles can be synthesized from synthetic or natural polymers. One team has developed poly(carboxybetaine) (PCB)-based zwitterionic nanoparticles (MCPZFS NP). These nanoparticles decrease microglia priming by lowering the levels of pro-inflammatory mediators and contributing to brain-derived neurotrophic factor (BDNF) secretion [81]. They also enhance Aβ recruitment to microglia, contributing to improved Aβ phagocytosis [81]. Beyond the action on microglia, these nanoparticles can also play on Aβ loading, neuronal damage, memory deficits, and neuroinflammation in APPswe/PS1dE9 mice [81]. Poly(lactic-co-glycolic acid) (PLGA), polyethylene glycol (PEG), and lipid chains as building blocks were used to synthesize 200 nm spherical polymeric nanoconstructs (SPNs) and 1000 nm discoidal polymeric nanoconstructs (DPNs). SPNs are more rapidly absorbed than DPNs and were used to encapsulate curcumin in the PLGA core. These curcumin-loaded nanoparticles decrease the production of proinflammatory cytokines-IL-1β, IL-6, and TNF-α in amyloid-β fiber-stimulated macrophages [83]. The α-M, previously used in a lipid nanoparticle, was encapsulated in the core of poly(ethylene glycol)-poly(l-lactide) (PEG-PLA) nanoparticles [NP(α-M)] [84]. This nanoformulation reduced Aβ deposition in AD and attenuated neuroinflammatory responses by microglia (using the BV-2 line) [84]. Apart from these effects on microglia, nanoencapsulation has improved the biodistribution and clearance of these molecules [84]. PLGA nanoparticles were used to deliver SurR9-C84A, a survivin mutant belonging to the inhibitors of the apoptosis protein family [85]. For this study, neuron monocultures and co-cultures of neurons and THP-1 (monocytes/macrophages) were used. These cultures were treated with LPS or β-amyloid to mimic the pathological inflammatory conditions of AD. Following this stimulation by LPS or β-amyloid, a decrease in THP-1 secretions was observed by the use of these nanoparticles [85]. This inhibition of secretions decreased the neuronal cell death induced by them.

Amphiphilic sugar-based molecules (AM) derived from mucic acid were synthesized to exhibit high affinity to scavenger receptors, allowing internalization of α-synuclein at the microglia [86]. Internalization of monomeric α-synuclein and formation of intracellular α-synuclein oligomers were decreased in microglial cells treated with these amphiphilic molecules. Following this observation, the antioxidant poly(ferric acid) was added to the core of these amphiphilic molecules by performing nanoprecipitation [86]. Microglial cells treated with these nanoparticles and stimulated by α-synuclein saw a decrease in their activation as well as in the neurotoxicity induced by α-synuclein aggregated at the level of microglia [86]. In vivo, the activation of microglia is also decreased after injection of these nanoparticles in the substantia nigra of mice stimulated by fibrillar α-synuclein. Targeting the receptors responsible for α-synuclein entry into microglia and adding an antioxidant may represent an interesting therapeutic approach via nanotechnology [86].

Polylactic acid (PLA)-coated mesoporous silica nanoparticles were loaded with resveratrol, which exhibits antioxidant activities among others. The PLA coating protects the resveratrol and prevents its systematic release. In the presence of oxidative stress, PLA is degraded and resveratrol can be released. Following this release, resveratrol was able to effectively reduce the activation of microglia cells stimulated by phorbol-myristate-acetate or lipopolysaccharide [87].

#### 6.1.4. Cell-Derived Nanoparticles

Extracellular vesicles are membrane-containing vesicles from the endocytic pathway or plasma membrane, released into the extracellular space by virtually all cells. Three types of extracellular vesicles exist: (a) exosomes, the smallest vesicles (30–150 nm), derived from the inward budding of multivesicular bodies; (ii) microvesicles, or ectosomes (50 nm–1 μm), result from outward budding of the plasma membrane, released under physiological conditions or in response to specific stimuli; and (c) apoptotic bodies (50 nm–5 μm), which are produced by cells undergoing apoptosis.

Exosomes contain cellular proteins, lipids, nucleic acids, mRNAs, and microRNAs (miRNAs) from host cells (Figure 3). Exosomes can be internalized by cells and functionally modify the cells that internalize them. Exosomes can be derived from activated or non-activated cells or loaded with therapeutic molecules to be used as cargo.

The most commonly used cells for the use of exosomes as therapeutic cargo are mesenchymal stromal cells (MSCs). Exosomes derived from MSCs or MSCs preconditioned by hypoxia (increased miR-21 expression) were systemically administered to APP/PS1 transgenic mice mimicking AD [88]. In both cases, the use of MSC-derived exosomes or hypoxia-preconditioned MSCs had positive effects. Administration of exosomes from hypoxia-preconditioned MSCs improved memory and learning abilities; decreased plaque deposition and Aβ levels; increased expression of growth-associated protein 43, synapsin-1, and IL-10; and decreased levels of GFAP, Iba-1, TNF-α, IL-1β, and activation of STAT3 and NF-κB [85]. The use of these exosomes would correct synaptic dysfunction as well as inflammatory responses, which would lead to improvement of cognitive decline observed in AD via miR-21 signaling [88]. Human umbilical cord mesenchymal stem cells (hucMSC-exosomes) were injected into mouse models of AD and were found to improve cognitive decline and decrease Aβ deposition [89]. These hucMSC-exosomes also modulated microglial activation with a decrease in the number of activated microglia and a shift toward an M2 anti-inflammatory profile (increase in IL-10 and TGF-β cytokines) with an increase in Aβ-degrading enzymes [89]. Brain perfusion of neuroblastoma-derived exosomes may mediate Aβ clearance in an AD mouse model. Indeed, Aβ peptides can be taken up and transported by exosomes for presentation to microglia, resulting in their degradation [90,91].

Exosomes can also be loaded with a therapeutic molecule. The exosomes can be passively incubated with the therapeutic molecule, followed by purification. Curcumin was incubated at 22 °C for 5 min with exosomes, and the mixture was then effectively administered to the brain by intranasal route [92,93]. This decreased inflammation via microglia targeting [92,93]. Other exosome loading strategies exist, such as electroporation, incubation at room temperature, permeabilization with saponin, freeze/thaw cycles, sonication, and extrusion, and have been tested in neurodegenerative diseases but without microglia targeting or in other pathologies like cancer.

Exosomes are able to modulate the inflammatory response and phagocytosis activity of microglia and can be considered as a very interesting possibility in therapy.

#### 6.1.5. Antioxidant Nanoparticles

Nanoparticles could also be synthesized directly from an antioxidant like quercetin. Authors have obtained quercetin nanoparticles with a very heterogeneous size ranging from 520 to 750 nm. In a model of AD, these quercetin nanoparticles were able to reduce neuronal damage, decrease the formation of amyloid plaques and neurofibrillary tangles, and modulate the activity of microglia [94].

In the context of PD, a nanoparticle (NP) formulation containing two polyphenol antioxidants, tannic acid (TA) and a ferulic acid diacid molecule, was proposed. These antioxidant nanoparticles inhibited α-synuclein fibrillation and lowered intracellular α-synuclein oligomerization in the BV-2 microglial line subjected to a high concentration of extracellular α-synuclein, thereby ameliorating microglial oxidative stress [95]. Microglial activation is also reduced with a modulation of the production of the pro-inflammatory cytokines TNF-α and IL-6 [95].

### 6.2. Dendrosomal Nanoparticles

An Iranian team used the dendrosome, a neutral, amphipathic, and biodegradable nanomaterial, to transport a molecule of interest such as curcumin. In a cuprizone-induced model of MS, these curcumin-loaded dendrosomes suppressed the accumulation of microglia and astrocytes, highlighting their possible use in therapy [96].

### 6.3. Dendrimers and Other Dendritic Polymers

Dendritic polymers belong to the synthetic polymers with linear, cross-linked, and branched polymers. Among these are the dendrimers which are obtained after generational synthesis (Figure 3), resulting in the formation of theoretically monodisperse structures with a narrow molecular weight distribution. In contrast to dendrimers, hyperbranched polymers are polydisperse, with a broad molecular weight distribution. The third members are dendrigrafts and have a configuration that shares commonalities between dendrimers and hyperbranched polymers (Figure 3).

Dendrimers, which are synthetic molecules with a tree-like structure, can be constructed by different methods: the hyperbranched structure can be built from the core, layer by layer, or by attaching dendrons to a central core [97,98]. Among the best-known dendrimers are poly(amidoamine) (PAMAM), poly(propylene imine) (PPI), phosphorus, and dimethylolpropionic acid-based dendrimers. One team has developed a dendrimer that targets the mitochondria (using triphenyl-phosphonium (TPP)) and delivers the antioxidant N-acetylcysteine (NAC):mitochondrial targeting hydroxyl PAMAM dendrimer-drug construct (TPP-D-NAC) [99]. The authors had already shown in previous studies that this PAMAM polymer was able to cross the BBB by selectively targeting activated microglia/macrophages and was able to deliver NAC to these cells (dendrimer D-NAC) [100]. These TPP-D-NAC dendrimers show preferential targeting to mitochondria (but not only) of activated microglial/macrophage cells. They showed superior efficacy in terms of oxidative stress inhibition to dendrimers developed from NAC but without organelle targeting (D-NAC) and to NAC used alone [99]. Concerning the dendrimers, the functionalization of the surface is an important factor to take into account, as it can play on the toxicity of the molecule [101]. Peroxisome proliferator-activated receptor (PPAR) α and PPARγ agonists can switch microglia from an M1-like to an M2-like phenotype. Because hydroxyl-terminated polyamidoamine dendrimers cross the altered BBB at the site of neuroinflammation and accumulate in activated microglia, they are conjugated with a PPARα/γ dual agonist [102,103]. The dual agonist dendrimer-PPARα/γ conjugate (D-tesaglitazar) induced the following: (a) an “M1 to M2’’ phenotype change, (b) a decrease in reactive oxygen species secretion, (c) an increase in the expression of phagocytosis and enzymatic degradation genes of pathogenic proteins, and (d) an increase in phagocytosis of β-amyloid [104]. 

Dendrigrafts have also been used to deliver treatment to the brain. Caspase-3 is involved in cell death and inhibiting it could help prevent the progression of neurodegenerative diseases and, in this study, PD. For this purpose, RNA interference was used as well as a vector constituted by dendrigraft poly-L-lysines, on which a peptide glycoprotein of the rabies virus with 29 amino acids was bound, allowing it to cross the BBB by transcytosis mediated by a specific receptor [105]. Plasmid DNA encoding the short hairpin RNA of caspase-3 was complexed with this vector to give nanoparticles. Injection by weekly intravenous administration of the nanoparticles reduced the levels of activated caspase-3, which decreased dopaminergic neuronal loss in the brains of rats with PD [105]. In addition, the rat model was obtained by treating them with rotenone, which increases TNF-α and NO levels in the brain. The use of these nanoparticles reduced the levels of TNF-α and NO in the brain [105]. These nanoparticles would thus have an anti-inflammatory effect on the microglia according to this study [105].

### 6.4. Quantum Dots

Quantum dots are fluorophores constituted by clusters of atoms at the nanometric scale containing a few hundred to a few thousand atoms of a semiconductor material wrapped by an additional semiconductor layer to improve the optical properties of the material (Figure 3). Quantum dots have been successfully used in immunocyto- and histochemistry, in flow cytometry, and in confocal microscopy [106,107].

Amino(polyethylene glycol)-2000 molybdenum disulfide quantum dots conjugated to (3-carboxypropyl)triphenyl-phosphonium bromide (TPP-MoS2 QDs) have been fabricated and tested in mouse models of AD [108]. These TPP-MoS2 QDs have the ability to cross the BBB and target mitochondria with TPP. These TPP-MoS2 QDs are able to induce a change in the phenotype of the microglia from pro-inflammatory M1 to anti-inflammatory M2 [108]. This contributes to the removal of Aβ aggregates [108]. 

## 7. Conclusions

DHA has shown cytoprotective effects on microglial cells but also on microglia activation in vitro and in vivo in animal models. In clinical studies, positive effects on the development of neurodegenerative diseases have been observed, but some conclusions are divergent and require further investigation, since DHA has a systemic and not a targeted release at the brain level. This may lead to the use of nanoparticles that could allow a release at the brain level of DHA and improve its effectiveness at the level of the microglia, thus leading to better management of neurodegenerative diseases associated with neuroinflammation.

## Figures and Tables

**Table 1:** In vitro data on the effect of DHA on cell death, oxidative stress, and inflammation.

Experimentation Type	Target	DHA Forms	Concentration or Dose	Effects	References
BV-2 cells treated with 7-ketocholesterol	Cell death oxidative stress	Chemical form	12 µM	Protection against cell death and oxidative stress	[15]
BV-2 cells	Cell death		200 µM	Induction of cell death (pyroptosis)	[16]
BV-2 cells treated with oligomeric amyloid-β peptide	Oxidative stress		10 µM	Reduce oxidative stress (involving Nrf2/HO-1)	[18]
BV-2 cells treated with LPS			1.25–10 μM	Enhance Nrf-2/HO-1 signaling	[19]
			10 µM DHA + quercetin (2.5 µM)		[20]
BV-2 cells treated with interferon-γ	Inflammation		30 µM	Reduce expression of proteins implicated in inflammation	[21]
BV-2 cells and MG6 cells stimulated with LPS			100 µM DHA and 100 µM EPA	Inhibition of inflammation by involving SIRT1	[22]
BV-2 cells stimulated with LPS			30 µM	Decrease cytokine expression	[17,23]
Primary cultures of mice microglial cells stimulated with LPS			20 µM to 80 µM	Reduce NO and TNF-α release, modulation of phenotypic polarization of microglia	[24]
Human CHME3 microglial cells exposed to Amyloid-β42			0.1 to 1 µM	Decrease pro-inflammatory cytokine production, induction of a shift in phenotype away from pro-inflammatory M1 activation	[25]
Rat glial primary cell cultures, LPS/ IFN-γ stimulation			100 µM	Regulation of the pro-inflammatory response	[26]
EOC20 microglia cells treated by Polyinosinic-Polycytidylic acid or 10 μg/mL Imiquimod			50 µM	Reduction of the production of cytokines TNF-α and IL-6	[27]
Primary neuron/glia rat primary cultures infected with Japanese Encephalitis virus			25 or 50 µM	Increase of neurotoxin cytokines production	[28]
BV-2 cells activated with LPS and IFN-γ		Triglyceride forms	20 µM	Reduce IL-6 and TNF-α production	[29]
BV-2 cells treated with LPS		N-docosahexaenoyl dopamine (DHDA)	2 µM	Decrease IL-6 and CCL-20 production	[30]
Primary cultures of rat microglia and BV-2 cells treated with LPS		Synaptamide	1–100 nM	Suppression of LPS-induced TNF-α and iNOS mRNA expression	[31,32]

**Table 2:** In vivo data on the effect of DHA.

Experimentation Type	Target	DHA Forms	Concentration or Dose	Effects	References
Injection of LPS intraperitoneally (i.p.) or in brain of C57Bl/6J mice	Inflammation	Synaptamide, endogenous metabolite derived from docosahexaenoic acid	5 mg/kg, i.p	Decrease of TNF-α, IL-1β, IL-6, iNOS, and CCL2 mRNA involving orphan adhesion G-protein-coupled receptor 110 (GPR110)	[32]
C57Bl/6J mice intraperitoneally injected with LPS		Fish hydrolysate supplement	143 µg in 150 µL of fish hydrolysate supplement/day	Reduce expression of TLR4, cytokines (IL-6, TNF-α, IL-1β,), CCL2, and IκB	[34]
		Chemical form	10 mg/day	Reduce IL-6 expression	[34]
C57Bl6/J mice injected with LPS (i.p.).		1-palmitoyl,2-docosahexaenoyl-PC (PC-DHA)	4.33 μg/g of mouse	Decrease IL-6 production	[23]
Piglets		Herring oil	32.30% W/W total fatty acids	No attenuation of the LPS induced inflammation	[35]
C57Bl/6 male mice with intracerebroventricular LPS injections		Fish oil	1.4% of total fatty acids	No change in the expression of pro-inflammatory genes	[36]
Triple-transgenic mouse model of AD		Neuroprotectin D1 (organic synthesis)	50 nM	Downregulation of Aβ42-induced expression of COX-2, TNF-α and B-94	[37]
Male C57BL/6 mice with intracerebroventricular amyloid-β infusion (AD model)		Fish oil	1.5% of total fatty acid	Decrease some elements of the inflammatory response	[38]
Male albino Swiss mice, administration of AlCl_3_ (20 mg/kg) intragastrically (i.g.) then an intraperitoneal injection with D-gal (120 mg/kg) (AD model)		Chemical form	200 mg/kg	Downregulation of TNF-α expression	[39]
TgCRND8 mice (AD model)		Whole-food diet contained skinless freeze-dried Atlantic salmon and a proprietary mixture of powdered, freeze-dried vegetables and fruits	0.246% of DHA (wt/wt) in a whole-food diet	Increase TNF-α expression	[40]
Male Tg2576 (APPswe) transgenic mice (AD model)		Chemical form	50 mg/kg body weight	Microglial activation	[41]
Male C57/BL6 mice fed for 5 weeks with a diet containing 0.2% cuprizone (MS model)		Regular diet supplemented with n-3 PUFAs	DHA + EPA, 15 g/kg	Suppression of the increase of M1-associated genes and increase of the expression of M2-associated genes	[24]
Female C57BL/6J mice immunization with >95% pure synthetic MOG35-55 peptide (MS model)	Development of the pathology	TG-DHA obtained by enzymatic synthesis	250 mg/kg/day	Improve clinical score	[29]
C57BL/6J female/EAE model (MS model)		Phospholipid-DHA 0.3% or 1% and triacylglycerol-DHA	0.3 or 1% either 0.48 or 1.6 mg DHA/g body weight/day respectively	Reduce EAE onset and severity	[42]

**Table 3:** Clinical trials involving DHA.

Experimentation Type	DHA Forms	Concentration or Dose	Effects	References
AD Patients	Fish oil	1 g/day of fish oil (30 mg DHA, 90 mg EPA)	Slowing down AD	[47]
AD Patients	Capsule EPAX 1050TG	four capsules (One capsule: 430 mg DHA and 150 mg EPA)	Induction of DNA hypomethylation in blood cell, can be used as treatment in AD	[48]
AD patients		2.3 g of omega-3 fatty acid	Positive correlation between plasma levels of omega-3 fatty acids and cognitive functions	[49]
Peripheral blood mononuclear cells treated with the Aβ40 peptide	Capsule EPAX 1050TG		Prevention of the reduction of specialized proresolving mediators (lipoxin A4 and resolvin D1) released from PBMCs	[50]
Moderate AD patients	Capsule EPAX 1050TG	four capsules (One capsule: 430 mg DHA and 150 mg EPA	No clear effect on oxidative stress but potential role in immunoregulation	[51]
AD patients	Capsule EPAX 1050TG	four capsules (One capsule: 430 mg DHA and 150 mg EPA	Increase in eicosapentaenoic acid (EPA), DHA, and total n-3 FA levels in cerebrospinal fluid	[52]
			Increase plasma levels of transthyretin which could influence Aβ peptide deposits in the brain	[53]
Peripheral blood mononuclear cells of AD patients			Regulation of genes involved in inflammation regulation, neurodegeneration, and in ubiquitination processes	[54]
LPS-stimulated peripheral blood mononuclear cells			Anti-inflammatory effects, reduction in the release of IL-1β, IL-6, and granulocyte colony-stimulating factor	[55,56]
Cognitive impairment: no dementia and AD patients	Capsules	625 mg DHA and 600 mg EPA	No beneficial effect on cognition and mood	[57]
Mild or moderate AD patients	Algal origin	2 g of capsule containing 15% to 55% DHA	No slowdown in cognitive decline	[58]
Mild or moderate AD patients	Capsule EPAX 1050TG	four capsules (One capsule: 430 mg DHA and 150 mg EPA	No effect on neuropsychiatric symptoms, possible positive effects on depressive symptoms in non-ApoEω4 carriers and on agitation symptoms in ApoEω4 carriers	[59]
Patients with organic brain damage or mild cognitive impairment	Aravita capsules	40 mg/capsule of arachidonic acid and DHA	Significant improvements in memory	[60]
Spinocerebellar ataxia 38	Algal oil	600 mg/day	Improvement in clinical symptoms and no degradation of neurophysiological parameters	[61,62]

## Data Availability

Not applicable.
